# Calibration of Elasto-Magnetic Sensors on In-Service Cable-Stayed Bridges for Stress Monitoring

**DOI:** 10.3390/s18020466

**Published:** 2018-02-05

**Authors:** Carlo Cappello, Daniele Zonta, Hassan Ait Laasri, Branko Glisic, Ming Wang

**Affiliations:** 1 Department of Civil, Environmental and Mechanical Engineering, University of Trento, 38122 Trento, Italy; 2 Department of Civil and Environmental Engineering, University of Strathclyde, Glasgow G1 1XQ, UK; daniele.zonta@strath.ac.uk; 3 Faculty of Sciences, Ibn Zohr University, 80000 Agadir, Morocco; hassan.or@hotmail.com; 4 Department of Civil and Environmental Engineering, Princeton University, Princeton, NJ 08544, USA; bglisic@princeton.edu; 5 Department of Civil and Environmental Engineering, Northeastern University, Boston, MA 02115, USA; mi.wang@northeastern.edu

**Keywords:** calibration, elasto-magnetic sensors, cable-stayed bridge, structural health monitoring, vibration test

## Abstract

The recent developments in measurement technology have led to the installation of efficient monitoring systems on many bridges and other structures all over the world. Nowadays, more and more structures have been built and instrumented with sensors. However, calibration and installation of sensors remain challenging tasks. In this paper, we use a case study, Adige Bridge, in order to present a low-cost method for the calibration and installation of elasto-magnetic sensors on cable-stayed bridges. Elasto-magnetic sensors enable monitoring of cable stress. The sensor installation took place two years after the bridge construction. The calibration was conducted in two phases: one in the laboratory and the other one on site. In the laboratory, a sensor was built around a segment of cable that was identical to those of the cable-stayed bridge. Then, the sample was subjected to a defined tension force. The sensor response was compared with the applied load. Experimental results showed that the relationship between load and magnetic permeability does not depend on the sensor fabrication process except for an offset. The determination of this offset required in situ calibration after installation. In order to perform the in situ calibration without removing the cables from the bridge, vibration tests were carried out for the estimation of the cables’ tensions. At the end of the paper, we show and discuss one year of data from the elasto-magnetic sensors. Calibration results demonstrate the simplicity of the installation of these sensors on existing bridges and new structures.

## 1. Introduction

Structural health monitoring (SHM) systems have gained rapid progress with the aid of advanced technologies in sensing, data communication and data analysis. They provide relevant information on structural behavior and are particularly important for early damage detection, reliability evaluation and residual capacity determination. Unnecessary periodical inspections can be avoided leading to a reduction of maintenance costs by using permanent monitoring systems. Nowadays, monitoring systems have been widely accepted and used to check the structural health and condition of civil infrastructure. More and more large-scale monitoring systems have been designed and implemented in existing structures and during the construction stages. However, the application of monitoring systems on existing structures can be a challenging and costly task. Many of these structures are cable-stayed or cable-suspended bridges. In these structures, the cables are the fundamental components that guarantee the overall structural safety. Therefore, permanent monitoring of cable stress is of great importance for assessing the health of such bridges. Measuring stress in cables during the service life of bridges can be extremely challenging if we use traditional methods. Elasto-magnetic (EM) sensors use a new low-cost technology for measuring the actual stress in ferromagnetic materials such as that of steel wires, strands and steel bars. They are considered to be a promising tool owing to heir non-destructive and non-contact properties, which also include corrosion resistance and long service life [1,2].

The fundamental principle of EM sensors is the magneto-elastic phenomenon of the ferromagnetic material. The magnetic properties of a ferromagnetic material change under the application of stress and under the influence of temperature. Thus, it is possible to measure the stress applied to a ferromagnetic material by knowing the relationship between magnetic permeability and stress. This relationship can be derived from the calibration process. The first studies about the magneto-mechanical effect were made in the 19th Century. Later studies discussed the theoretical background of the magnetization process in ferromagnetic metals and its application to the magneto-mechanical effect [3,4]. In recent years, many investigations have been devoted to developing, installing and improving the performance of EM sensors. In 1996, Kvasnica and Fabo [5] designed a microcomputer-based instrument for the magnetic measurement of mechanical stress in low-carbon steel wires. In 1998, Wang et al. introduced the concept of stress monitoring in the cables of cable-stayed bridges by using EM sensors [6] and confirmed, in 2000, that EM technology can provide adequate accuracy and reliability to monitor the actual stress of tendons and cables [7]. The reliability of EM sensors in measuring the actual stress of cables and reinforcement bars has been shown by several literature sources [1,8]. Others have applied EM sensors to determine the fatigue state in the ferromagnetic steels [9]. In 2003, Cajko discussed a pulse variant method for measuring the incremental permeability, which improves information and reduces the specimen heating caused by the traditional techniques [10]. In 2004, numerous experiments were performed by Singh et al. on ferromagnetic steels at different temperatures and for different cross-sections, in order to test the accuracy of EM sensors in corrosion evaluation [11]. Rumiche et al. have reported that EM sensors can possibly be used as a reliable non-destructive tools for the detection of corrosion in the early stages of carbon steels [12]. Furthermore, Park et al. have proposed a monitoring technique that enables a cable-climbing robot to detect cross-sectional losses [13]. In 2008, Wang verified the capability of EM sensing technology for long-term structural health monitoring of the external tendons of a double-box girder bridge [14]. In the same year, Tang et al. designed a new EM sensor that performs temperature compensation in a wide temperature range [15]. The use of EM sensors for the detection of creep in ferromagnetic materials was examined by Polar et al., in 2010 [16]. In the same year, Cao and Wang studied structural effects using data collected by EM sensors [17]. In 2011, Duan et al. developed a smart elasto-magneto-electric sensor for stress monitoring in railway infrastructures [18]. In 2012, Duan et al. proposed a magneto-electric sensing unit to replace the secondary coil of conventional EM sensors [19].

In this work, we present how we can instrument in-service cable-stayed bridges with an EM sensor network, in order to continuously monitor the actual stress of its cables and guarantee the structural reliability. EM sensors are usually prefabricated and installed on bridge cables during the construction stages [1,20]. In this situation, the calibration can be carried out by loading and unloading the cables while recording the temperature. However, in our case, the sensors had to be installed two years after the bridge construction, and the owner could not allow the cables to be unloaded in order to perform calibration tests. In this contribution, we demonstrate a low-cost calibration and installation procedure for monitoring the tension of stay cables using EM sensors. This procedure is suggested for monitoring stresses in in-service cable-stayed bridges.

**Figure 1:**
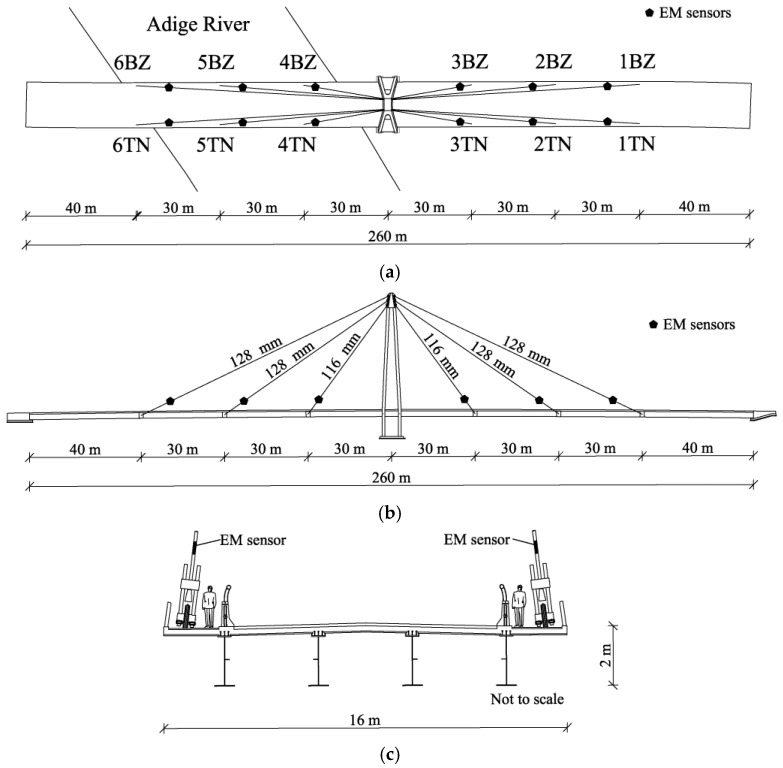
Adige Bridge: (**a**) plan view with sensor names (1TN–6TN, 1BZ–6BZ); (**b**) lateral view; (**c**) cross-section of the deck.

The monitored structure, presented in Figure 1, is a cable-stayed bridge spanning Adige River, 10 km north of the town of Trento, Italy. It is a statically indeterminate structure, having a composite steel-concrete deck of a length of 260 m overall supported by 12 stay cables, six per deck side. The deck cross-section consists of four “I” section steel beams of a depth of 2 m carrying a 25 cm-thick concrete slab. The deck is anchored to a cable every 30 m. The bridge tower is made of four pylons; it is 45 m high and is located in the center of the bridge. The stay cables are full-locked steel cables of diameters of 116 mm–128 mm, and they are designed for operational loads between 5000 and 8000 kN. Structural redundancy, possible relaxation losses and an as-built condition that are different from design suggest that long-term load redistribution between cables can be expected [21].

This paper is organized as follows. In Section 2, we introduce the physical principle of an EM sensor and justify the need for calibration. Next, we discuss the calibration process, which is divided into two phases: one in the laboratory and the other one on site. Section 3 is devoted to the laboratory calibration, where a sensor was built around a segment of cable, identical to those of Adige Bridge, and loaded up to 9000 kN. The response of the sensor at various load levels is compared with the load applied by the machine. Section 4 discusses the installation process and presents the in situ calibration, during which we carried out vibration tests in order to estimate the tension force of the actual bridge cables. Next, we assess the sensor accuracy and present one year of data recorded from the EM sensors after calibration. Finally, Section 5 outlines the conclusions.

## 2. Sensor Physical Principle

When a magnetic field *H* is applied to a medium, the resulting magnetic flux density *B* is related to *H*, given the magnetic permeability *μ* of the medium. The value of *μ* measures how easily a magnetic field can traverse a medium. For ferromagnetic materials, this relationship is nonlinear and hysteretic. Thus, we normally refer to the incremental permeability, which is the ratio between the incremental changes in the two magnitudes:

(1) $$\mu =\frac{\Delta B}{\Delta H}.$$

**Figure 2:**
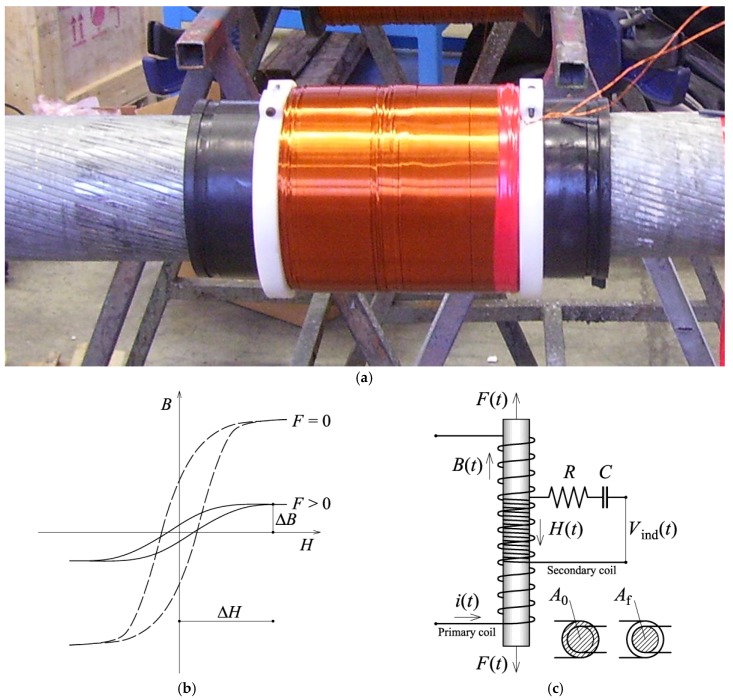
EM sensors: (**a**) picture of an EM sensor; (**b**) typical hysteresis loops for stressed and non-stressed ferromagnetic materials; (**c**) measurement principle.

The magnetic properties of a ferromagnetic material are altered with the application of stress. The magnetic strain energy *E_σ_* is related to stress *σ* and the angle *θ* between the direction of the applied stress and the magnetization vector, according to the equation:

(2) $$E_{\sigma }=\frac{3}{2}\lambda_{s}\sigma \sin^{2}\theta ,$$

where *λ*_s_ is the bulk magneto-restriction strain induced when the sample is magnetized to saturation magnetization [8]. Equation (2) shows that, in order to minimize *E_σ_*, *θ* has to change as *σ* changes. In other words, the random magnetization field that characterize the specimen before the magnetic field is applied rotates because of the variation of *σ*, making a magnetization in another direction more or less difficult. The measured permeability changes as a result. Hence, the cable stress state can be obtained from experimental measurements of the magnetic permeability. The easiest way to achieve this goal is through the principle of magnetic induction: by magnetizing the material using two solenoids. An EM sensor consists of two coils wound round the tensioned cable, as depicted in Figure 2a. In order to make a measurement, the cable is subjected to a pulsed magnetic field *H* generated by passing a pulsed current through the primary coil. Long measurements are avoided because they would increase the temperature of the sensor and consequently cause errors during the reading. The changes in the flux density *B* produce an output voltage *V*_ind_(*t*) across the secondary coil around the cable. The induced voltage *V*_ind_(*t*) allows the magnetic properties to be sensed and deduced through Faraday’s law:

(3) $$V_{\mathrm{ind}}\left(t\right)=-N\left[A_{f}\frac{dB\left(t\right)}{dt}+\left(A_{0}-A_{f}\right)\mu_{0}\frac{dH\left(t\right)}{dt}\right],$$

where *N* is the number of turns in the secondary coil, *μ*_0_ is the magnetic permeability of the free space and *A*_0_ and *A*_f_ are the cross-sectional areas of the secondary coil and the steel element (i.e., the cable), respectively. If the induced voltage is integrated in the time interval [*t*_1_, *t*_2_], the time-averaged voltage output on the secondary circuit is:

(4) $$V=-\frac{1}{t_{2}-t_{1}}\int_{t_{1}}^{t_{2}}V_{\mathrm{ind}}(t)dt,$$

or:

(5) $$V=\frac{1}{RC}NA_{f}\left[\Delta B+\left(\frac{A_{0}}{A_{f}}-1\right)\mu_{0}\Delta H\right],$$

where:

(6) $$\Delta B=\int_{t_{1}}^{t_{2}}\frac{dB}{dt}dt,\;\Delta H=\int_{t_{1}}^{t_{2}}\frac{dH}{dt}dt,$$

and *R* and *C* are the resistance and capacitance of the circuit shown in Figure 2c. If we take the same measurement without the ferromagnetic material, the resulting output voltage *V*_0_ is:

(7) $$V_{0}=\frac{1}{RC}NA_{0}\mu_{0}\Delta H.$$

By taking the ratio of (5) and (7), the permeability can simply be derived from the following equation:

(8) $$\frac{V}{V_{0}}=\frac{1}{\mu_{0}}\frac{A_{f}}{A_{0}}\frac{\Delta B}{\Delta H}+\left(1-\frac{A_{f}}{A_{0}}\right)=\mu_{r}\frac{A_{f}}{A_{0}}+\left(1-\frac{A_{f}}{A_{0}}\right),$$

where *μ*_r_ is the relative permeability of the ferromagnetic material:

(9) $$\mu_{r}=\frac{1}{\mu_{0}}\frac{\Delta B}{\Delta H}.$$

From Equations (8) and (9), we eventually obtain:

(10) $$\mu_{r}=1+\frac{A_{0}}{A_{f}}\left(\frac{V}{V_{0}}-1\right).$$

Equation (10) directly correlates the relative permeability *μ*_r_ of the steel core to the sensor output *V*. Since the output voltage is temperature and stress dependent, the sensor must be calibrated in order to measure only the stress of the steel rod.

## 3. Laboratory Calibration

The first calibration phase was carried out in laboratory conditions using segments of cables identical to those of Adige Bridge. The cables, with an EM sensor attached, were subjected to different values of load and temperature. The EM sensors used in this project were supplied by Intelligent Instrument Systems Inc (Burr Ridge, IL, USA). In order to install these sensors on the specimen, the primary coil and secondary coil were wrapped around the cable by a winding rig. Then, the measurements were obtained by recording the permeability variations of the steel core based on the voltage induced in the secondary coil. This voltage is sensitive to: (1) intensity of magnetization; (2) stress applied to the cable; (3) cable cross-section; (4) sensor manufacturing process; (5) temperature. Therefore, the quantification of permeability variations requires investigating the effect of each variable through the calibration process, which must be performed before using these sensors. The scope of the calibration is to provide the laws relating the sensor measurements to the stress applied to the cable and to compensate the effects of the other variables. If we take into account the effect of all parameters in Equation (8), we obtain:

(11) $$\mu_{r}(\sigma ,T,H)=1+\frac{A_{0}}{A_{f}}\left(\frac{V(\sigma ,T,H)}{V_{0}}-1\right),$$

where *σ* is the stress applied to the cable and *T* is the temperature at which the measurement was done.

First, it is necessary to eliminate the dependency of the sensor measurements on the magnetic field *H*. This process is experimentally completed by finding the magnitude of the excitation current that is necessary to make each sensor work. The relationship between the magnetic permeability and the stress of the cable is linear and stable provided the applied field *H* is equal to an optimum value [7]. In fact, the magnetic field *H* should be high enough to technically saturate the cable. The magnetic saturation is important to obtain the highest possible sensitivity and linearity. However, increasing the current generates heat, which worsens the performance. Thus, the input current needs to be optimized. In order to do so, the producer of the sensors performs a set of laboratory tests at various conditions, so that the best working range can be determined and the effects of *H* can be ignored in (11).

Next, the effects of temperature and stress on the relative permeability were analyzed using different stress and temperature levels. This investigation is necessary in order to identify the stress-permeability relation and compensate the influence of temperature. Since we are interested in the permeability variations related only to stress variations, the initial permeability of the cable at zero stress has to be subtracted from the measured one. This operation can simply be accomplished by solving:

(12) $$\Delta \mu_{r}^{\prime }\left(\sigma ,T,T_{0}\right)=\mu_{r}\left(\sigma ,T\right)-\mu_{r}\left(0,T_{0}\right).$$

By using Equation (12), we obtain:

(13) $$\Delta \mu_{r}^{\prime }\left(\sigma ,T,T_{0}\right)=\left[1+\frac{A_{0}}{A_{f}}\left(\frac{V(\sigma ,T)}{V_{0}}-1\right)\right]-\left[1+\frac{A_{0}}{A_{f}}\left(\frac{V(0,T_{0})}{V_{0}}-1\right)\right].$$

Then,

(14) $$\Delta \mu_{r}^{\prime }\left(\sigma ,T,T_{0}\right)=\frac{A_{0}}{A_{f}}\left[\frac{V\left(\sigma ,T\right)-V\left(0,T_{0}\right)}{V_{0}}\right],$$

where *T*_0_ is the baseline temperature at which *V*_0_ and the permeability at zero stress are measured.

The temperature effects can be mathematically excluded using the common experimental formula, which expresses the steel permeability variation due to temperature deviation from the baseline *T*_0_. The formula was experimentally tested by many researchers [22] and confirmed in this study:

(15) $$\Delta \mu_{r}^{\prime \prime }(T)=\alpha (T-T_{0}),$$

where *α* = d*μ*_r_/d*T* is the temperature sensitivity or temperature compensation coefficient.

By compensating the influence of temperature on the measured permeability, Equation (14) becomes:

(16) $$\Delta \mu_{r}\left(\sigma ,T,T_{0}\right)=\Delta \mu_{r}^{\prime }\left(\sigma ,T,T_{0}\right)+\Delta \mu_{r}^{\prime \prime }\left(T\right)=\frac{A_{0}}{A_{f}}\left[\frac{V\left(\sigma ,T\right)-V\left(0,T_{0}\right)}{V_{0}}\right]+\alpha \left(T-T_{0}\right).$$

**Figure 3:**
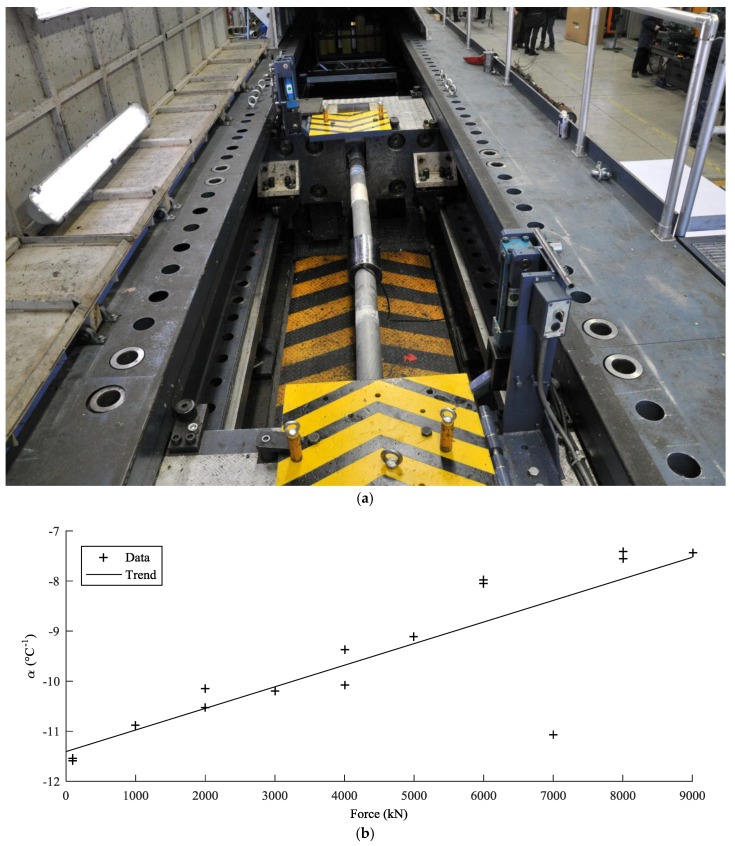
(**a**) View of an EM sensor on a 116-mm cable during laboratory calibration; (**b**) permeability sensitivity *α* (to temperature) for the cable of diameter 128 mm; the coefficient varies with the load level.

**Figure 4:**
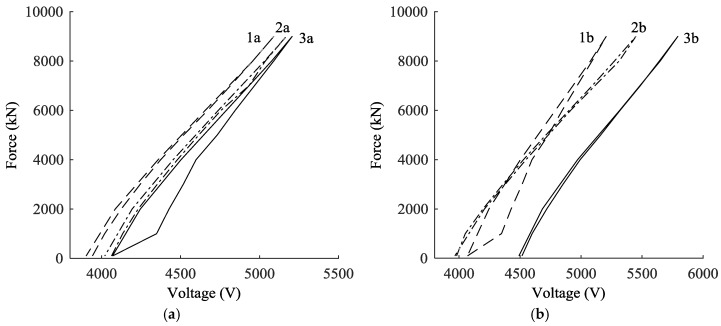
(**a**) different load-voltage calibration curves of the same sensor: the (3a) and (2a) lines are for different load cycles at temperature *T* = 27.2 °C, while the (1a) line was recorded at temperature *T* = 37.7 °C; (**b**) force to voltage ratio for three different sensors, after temperature compensation.

This equation relates the permeability variation due to the stress to the voltage output and temperature variation. By substituting the temperature-compensated relative permeability Δ*μ*_r_, the force *F* applied to the cable can be estimated by polynomial interpolation:

(17) $$F=C_{0}+\sum_{n=1}^{m}C_{n}\Delta \mu_{r}^{n}=C_{0}+\sum_{n=1}^{m}C_{n}{\left\{\frac{A_{0}}{A_{f}V_{0}}\left[V\left(\sigma ,T\right)-V\left(0,T\right)+\alpha \left(T-T_{0}\right)V_{0}\right]\right\}}^{n},$$

where *C_n_* are coefficients independent of temperature to be derived from the calibration process. In order to determine these coefficients, as well as the parameters *α* and *m*, the sensor response was measured under different load and temperature conditions. The load calibration provided *C_n_* and *n*, while temperature calibration provided *α*. During the load calibration, the cables, with the EM sensors attached, were connected to the tensing machine of Figure 3a, under constant temperature conditions. Then, the specimens were loaded and unloaded up to a force of 9000 kN, in steps of 1000 kN. After each load step, the sensor response was measured with at least three voltage readings (as suggested by the manufacturer) and compared to the force applied by the machine. Figure 3b shows that the temperature sensitivity of the permeability coefficient slightly depends on load. The permeability for the force of 7000 kN is an outlier and must be ignored. Figure 4a shows the relationship between the applied load *F* and the voltage *V*, for a cable of a diameter of 128 mm. Curves 3a and 2a are the responses of the first and second load cycles, at a temperature of 27.2 °C. It is clear that the relationship is not necessarily linear for every value of force. However, as long as the force is greater than 4000 kN, the relationship can be considered linear. Since the load applied to the cable of Adige Bridge is above 4000 kN, a linear equation was adopted for the force estimation. Moreover, the laboratory calibration showed that that the relationship curves obtained during several load cycles become more similar after the second cycle.

The temperature-permeability relationship was studied, and *α* was estimated by taking voltage readings at different loads for two different temperatures: 27.2 °C and 37.7 °C. Curves 1a and 2a of Figure 4a show an example for a cable with a diameter of 128 mm. The result indicates that the temperature modification does not affect the slope of the force-voltage curve *F-V*, but changes its position. It appears that the influence of temperature is to shift the curve *F-V* to a new position. This result is in accordance with Equation (15). A deep examination of the coefficient *α* reveals that it is not exactly constant, but changes slightly with the stress level: as is shown in Figure 4b, values from 0.011 °C^−1^ to 0.007 °C^−1^ have been identified at different load levels. Fortunately, the relationship is linear and can be taken into account in the calibration equation. The temperature sensitivity also depends on the cable diameter.

Based on the previous considerations, we can assume that the *F-V* relationship is linear:

(18) $$F=F_{0}+a\cdot \left(\left(V-V_{0}\right)-b\cdot \left(T-T_{0}\right)\right),$$

where *a* = d*F*/d*V* is the force to voltage slope and *b* = d*V*/d*T* is the voltage to temperature sensitivity. Equation (18) allows the calculation of the force applied to a cable, given its calibration coefficients *a, b*, the voltage response *V* at temperature *T* and the response *V*_0_ to a reference load *F*_0_ and temperature *T*_0_. Table 1 shows the mean and standard deviation of coefficients *α*, *a* and *b*, identified during the laboratory calibration (data of the third cyclic loading were used).

Further tests were carried out to verify the repeatability of measurements. Figure 4b shows the response of three different sensors manufactured in the laboratory on the same cable and using the same procedure. The curves are similar in the slope *a*, but exhibit very different shifts in position, making the installation of each sensor non-repeatable. Additional tests showed that the voltage-to-temperature sensitivity d*V*/d*T* and *α* are virtually independent of the manufacturing process. The scatter in the curve of slope *a* is relatively small and can be attributed to a bias error.

As a result of the laboratory calibration, a relationship of the type shown by Equation (18) is achieved for each combination of cable and sensor. However, due to the uncertainty in reference load *F*_0_, each sensor requires an on-site calibration after installation for at least one value of tension and temperature.

## 4. Installation, On-Site Calibration and Accuracy Estimation

**Figure 5:**
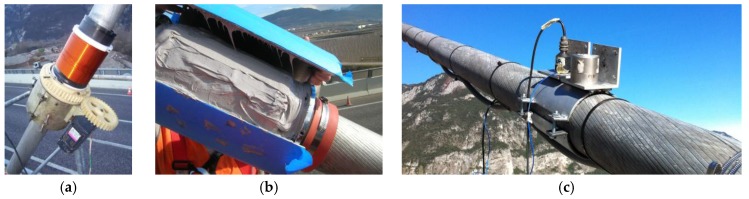
(**a**) Primary and secondary coil of EM sensors are wound around cables; (**b**) the two coils are protected by epoxy covers; (**c**) installation of accelerometers for in situ calibration.

After the laboratory calibration, 12 EM sensors were manufactured on site and installed on the 12 cables of Adige Bridge. The primary and secondary coils were wound around each cable by a winding machine, as shown in Figure 5a. After completing the winding procedure, temperature gages were attached, and electric cables were connected. The two coils were separated by plastic shells and protected by half-cylindrical epoxy covers (Figure 5b).

**Figure 6:**
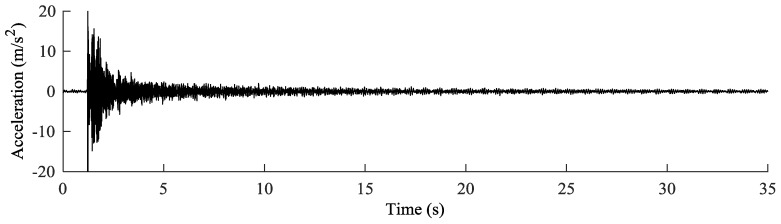
Data recorded by the accelerometers for cable 1TN (see Figure 1a).

**Figure 7:**
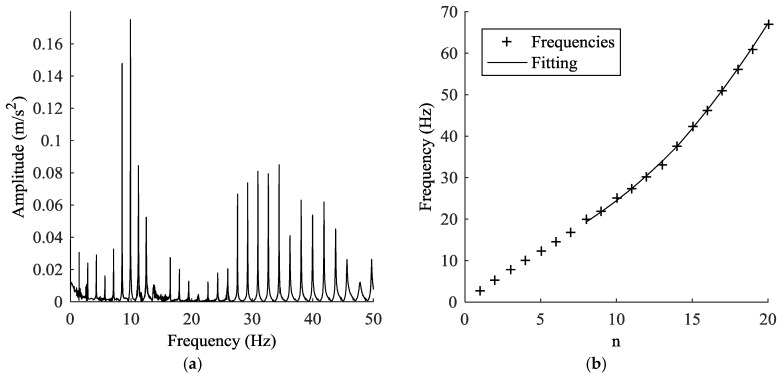
(**a**) Fast Fourier transform of the signal acquired on the cable 1TN and (**b**) comparison between the measured frequencies (points) and the theoretical trend (continuous line).

The in situ calibration was carried out without unloading the stay cables. The goal of this calibration was to define, for each cable, the offset *F*_0_ that the laboratory calibration showed to be dependent on the installation process. In order to estimate the reference loads of the cables of Adige Bridge without releasing the cables, vibration tests were performed. Two different accelerometers were applied near each other on each cable (Figure 5c). The connection was made by fastening a steel shell on the cable. Then, the accelerometers were glued to an aluminum plate, and the plate bearing the accelerometers was later screwed to the shell. The whole system was considered perfectly rigid. The test was carried out for each cable by recording its response to a hammer blow with a rate of 500 Hz. Figure 6 shows as an example the response of cable 1TN (Figure 1a). Next, frequency spectrums were obtained by implementing a fast Fourier transform (FFT). The frequency spectrum of cable 1TN is shown in Figure 7a. Despite the peak frequencies being clearly visible in the graphs obtained through the FFT, the natural frequencies were calculated by fitting the spectrums (Figure 7b) with the theoretical expression:

(19) $$q\left(f\right)=\sum_{n=1}\frac{A_{n}f^{2}}{\left|f_{n}^{2}-f^{2}-2\xi \cdot i\cdot f_{n}\cdot f\right|},$$

where *q* is the acceleration, *f_n_* are the peak frequencies, *f* is the frequency (the independent variable), *A_n_* are the acceleration at the peak frequencies, *ξ* is the relative damping and i^2^ = −1. In (19), parameters *A_n_*, *ξ* and *f_n_* were considered unknown, while the sum was carried out up to frequencies of about 45–50 Hz. This let us identify the natural frequencies *f_n_* shown in Table 2 with a precision of 0.02 Hz. After extracting the harmonic series of the signal, the tension was estimated using the following expression [23,24,25]:

(20) $$F=k{\left(\frac{2L}{n}\right)}^{2}f_{n}^{2}-{\left(\frac{\pi n}{L}\right)}^{2}EJ,$$

where *k* is the linear mass of the cable, *L* the cable length, *f_n_* the frequency of the *n*th harmonic, *E* the apparent Young’s modulus of the cable steel and *J* the moment of inertia of the cable cross-section. More specifically, we fitted the relationship between the experimental frequency *f_n_* and the harmonic order *n* using *F* as a parameter.

Despite this method considering only the bending effect and neglecting the effects of sag-extensibility, this approach is often used in practice due to its simplicity and speediness. For cable 1TN, the effect of parameter estimation is shown in Figure 7b. It was observed that the first seven modes deviate from the curve fitting obtained from the other modes. The reason may be attributed to the sag-extensibility effects, which mainly affect the lower modes. It has been proven by many studies that the utilization of higher modes provides better accuracy [25,26]. Thus, the first seven lower modes were neglected in this work.

While the vibration test was being performed, the voltage of the EM sensor installed on the vibrating cable was recorded together with its temperature value. Table 3 shows the acquired data.

**Figure 8:**
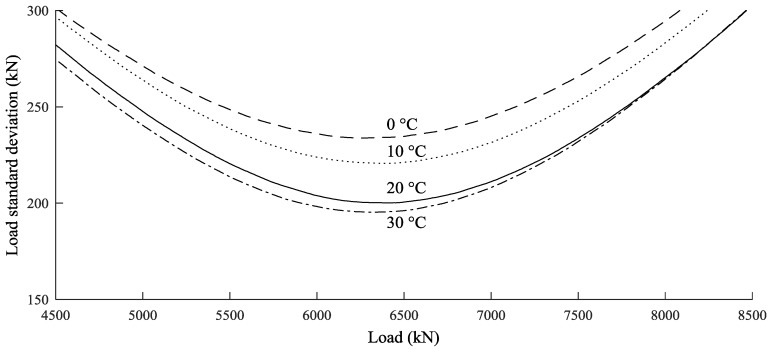
Standard deviation of load on cable 5TN estimated by Equation (17).

Experimentally, it has been found that the estimated loads have limited accuracy even when many harmonics are identified: the standard deviation of the baseline cable tension was 200–300 kN for cable tension varying from 4000 kN to 7000 kN. Furthermore, due to uncertainties in the coefficients *a* and *b*, the accuracy deteriorates when the conditions are different from those of calibration. Figure 8 shows the expected standard deviation for different values of tension and temperature simulated using Monte Carlo analysis [27]. The graph shows that the most significant source of error is the inaccuracy of the baseline measurement. Compared to this, the uncertainty in force sensitivity is not critical, while the temperature sensitivity can result in an additional error of 50 kN. Similar results were found for all the cables of Adige Bridge.

## 5. Monitoring Data

**Figure 9:**
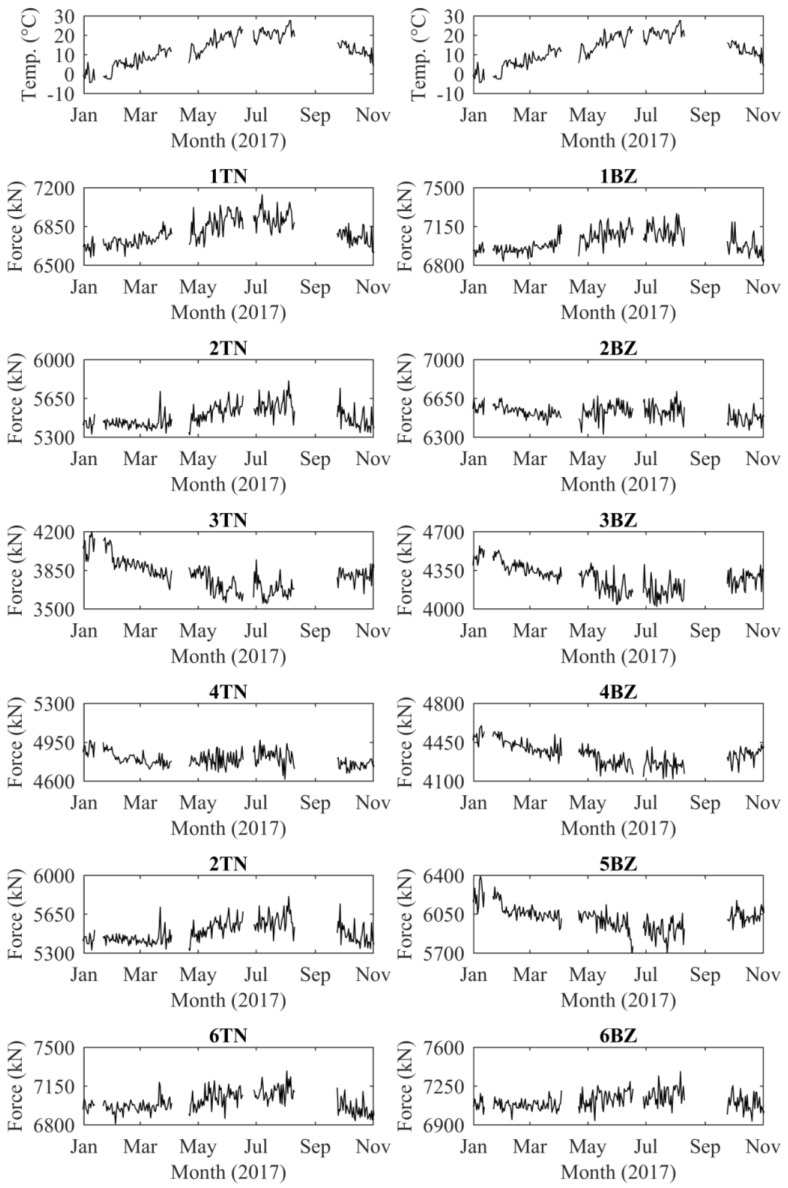
Calibrated monitoring data.

The in situ calibration of the force in each cable enabled the continuous acquisition of data from the monitoring system. The values of temperature and force acquired since 1January 2017 are shown in Figure 9. The effect of temperature on the sensors was removed from the data of Figure 9, while the effect of temperature on the structure was not. The figure shows, for each cable, one value per day, recorded in the early morning, at about 6:00 a.m. At this time, the temperature of the whole structure is approximately homogenous. However, the average daily temperature changes during the year. Since the structure is statically indeterminate, we expected different temperature effects on different cables due to load redistribution. Figure 9 proves that the force in the cables is severely affected by the temperature variations. The monitoring data show that the force of the cables anchored near the bridge shoulders (1TN, 1BZ, 6TN and 6BZ) increases with temperature, while the force of the cables anchored near the tower (3TN, 3BZ, 4TN and 4BZ) decreases with temperature. The explanation of this phenomenon is beyond the scope of this paper and will be made in future work. Nevertheless, the force recorded from the stay cables was compatible with the behavior predicted during the design of the structure and was in the range of the force obtained from vibration tests, which confirms the reliability of EM sensors.

## 6. Conclusions

In this contribution, we begin by providing a description of EM sensors for the monitoring of tension in ferromagnetic cables. Then, using the case study of Adige Bridge, we show the feasibility of monitoring the force in stay cables. We explain how calibration of EM sensors and installation on existing cables can be easily performed. One year of data from the EM sensors installed on Adige Bridge is presented at the end of the paper in order to show the reliability of the sensors. Our concluding remarks on the use of this technology can be summarized as follows.

The stability of the force-to-voltage and voltage-to-temperature sensitivity, which are not affected by the installation process, assert EM sensors’ applicability and eases the installation of EM sensors on existing structures.

Experimental results showed that two calibration stages are required: the first must occur in laboratory conditions, and the second is to be performed on site. The former is needed in order to define the force-to-voltage and voltage-to-temperature sensitivity; the latter is required to determine the offset in voltage, which depends on installation.

EM sensors can measure the real stress of a steel cable even when the zero-stress state of the cable is unknown. Actually, neither the laboratory calibration, nor the in situ calibration enabled us to measure the zero-stress state, because the force-to-voltage relationship was non-linear for small values of load.

However, in order to monitor the actual force of stay cables such as those of Adige Bridge, it is required to measure at least one value of real stress and the corresponding sensor response and temperature for each cable. In our case study, in order to obtain the tension of the existing cables, vibration tests were carried out.

For Adige Bridge, the precision of the force measurements was better than 200 kN, which is a relatively high value, but acceptable. This precision was mainly due to the inaccuracy of the baseline measurements (second calibration stage).

To sum up, EM sensors are a promising, simple and affordable tool for stress monitoring of steel structures. EM sensor technology enables simple and inexpensive installation on in-service bridges, without any change being made to the bridge structures. Moreover, it provides adequate accuracy and reliability for monitoring the actual stress of steel cables during the entire service life of civil structures.

## Figures and Tables

**Table 1:** Mean and standard deviation (StD) of coefficients *α*, *a* and *b* identified by laboratory calibration.

Cable Diameter (mm)	*α* (1/°C)		*a* (kN/V)		*b* (V/°C)	
	Mean	StD × 10^−3^	Mean	StD	Mean	StD
116	–8.79	1.40	6.35	0.34	–7.48	2.08
128	–9.27	2.42	6.20	0.66	–8.88	1.32

**Table 2:** First 16 frequencies in Hz obtained with FFT.

No.	1TN	2TN	3TN	4TN	5TN	6TN	1BZ	2BZ	3BZ	4BZ	5BZ	6BZ
1	–	1.913	2.568	2.698	1.827	1.419	1.459	1.923	2.618	2.689	1.892	1.447
2	2.641	2.659	5.201	5.372	3.770	2.857	2.914	3.845	5.217	5.349	3.777	2.889
3	3.964	3.825	7.782	8.059	5.644	4.264	4.360	5.768	7.808	8.015	5.653	4.318
4	5.280	5.730	10.107	10.561	7.512	5.669	5.781	7.625	10.222	10.509	7.521	5.732
5	6.590	7.585	12.181	12.774	9.285	7.089	7.227	9.494	12.398	12.725	9.293	7.185
6	7.901	9.416	14.384	14.934	11.006	8.500	8.680	11.270	14.941	14.847	11.065	8.613
7	9.176	11.205	16.813	17.459	12.540	9.878	10.080	12.875	16.834	17.251	12.604	10.012
8	10.388	12.799	19.639	20.293	14.400	11.189	11.438	14.545	19.456	20.104	14.314	11.344
9	11.590	14.441	22.360	23.277	15.985	12.479	12.790	16.316	22.660	23.044	16.024	12.651
10	12.774	16.212	25.553	26.431	17.986	13.695	14.074	18.303	25.812	26.170	18.010	13.991
11	14.015	18.223	28.856	29.737	20.040	14.946	15.447	20.387	29.022	29.411	20.102	15.168
12	15.256	20.273	32.152	33.184	22.190	16.417	16.842	22.560	32.324	32.752	22.194	16.614
13	16.647	22.435	35.594	35.856	24.370	17.926	18.346	24.788	35.766	36.200	24.382	18.127
14	18.074	24.654	39.083	40.719	26.680	19.467	19.921	27.061	39.246	39.743	26.633	19.676
15	19.511	26.899	42.119	44.537	28.946	21.034	21.506	29.384	42.837	43.370	28.893	21.263
16	21.019	29.205	46.878	48.485	31.262	22.640	23.143	31.741	46.217	47.068	31.245	22.880

**Table 3:** Forces from vibration tests and voltages acquired during the in situ calibration.

Cable	1TN	2TN	3TN	4TN	5TN	6TN	1BZ	2BZ	3BZ	4BZ	5BZ	6BZ
*F_i_* (kN)	6670	5400	4260	4930	6320	7030	6930	6600	4570	4600	6320	7120
*σ*_F,*i*_ (kN)	151	194	188	206	185	148	154	196	191	203	186	152
*V_i_* (V)	5189	5248	4631	4589	5368	5282	5374	5139	4480	4496	5303	5584
*σ*_V,*i*_ (V)	5	7	5	5	5	5	12	9	5	5	5	13
*T_i_* (°C)	23	31	26	22	13	14	30	26	21	18	17	15
*σ*_T,*i*_ (°C)	0.5	0.5	0.5	0.5	0.5	0.5	0.5	0.5	0.5	0.5	0.5	0.5

## References

[1] Sumitro S., Kurokawa S., Shimanoand K., Wang M.L. (2005). Monitoring based maintenance utilizing actual stress sensory technology. Smart Mater. Struct..

[2] Jarosevic A. (1998). Magnetoelastic method of stress measurement in steel. Smart Struct. NATO Sci. Ser..

[3] Jiles D.C., Atherton D.L. (1984). Theory of the magnetization process in ferromagnets and its application to the magnetomechanical effect. J. Phys. D Appl. Phys..

[4] Langman R. (1985). The effect of stress on the magnetization of mild steel at moderate field strengths. IEEE Trans. Magn..

[5] Kvasnica B., Fabo P. (1996). Highly precise noncontact instrumentation for magnetic measurement of mechanical stress in low-carbon steel wires. Meas. Sci. Technol..

[6] Wang M.L., Satpathi D., Koontz S., Jarosevic A., Chandoga M. Monitoring of cable forces using magneto-elastic sensors. Proceedings of the 2nd US-China Symposium Workshop on Recent Developments of Computational Mechanics in Structural Engineering.

[7] Wang L.M., Chen Z.L., Koontz S.S., Loyd G. Magnetoelastic permeability measurements for stress monitoring in steel tendons and cables. Proceedings of the SPIE Nondestructive Evaluation of Highways, Utilities, and Pipelines.

[8] Sumitro S., Jarosevic A., Wang M.L. Elasto-magnetic sensor utilization on steel cable stress measurement. Proceedings of the First Fib Congress, Concrete Structures in the 21th Century.

[9] Grimberg R., Leitoiu S., Bradu B.E., Savin A., Andreescu A. (2000). Magnetic sensor used for the determination of fatigue state in ferromagnetic steels. Sens. Actuators A.

[10] Čajko F. Pulse elasto-magnetic measurement of the cylindrical-shaped ferromagnetic specimens. Proceedings of the 9th International Workshop on Applied Physics Matter (APCOM 2003).

[11] Singh V., Lloyd G.M., Wang M.L. (2004). Effects of temperature and corrosion thickness and composition on magnetic measurements of structural steel wires. NDT E Int..

[12] Rumiche F., Indacochea J.E., Wang M.L. (2008). Detection and monitoring of corrosion in structural carbon steels using electromagnetic sensors. ASME J. Eng. Mater. Technol..

[13] Park S., Kim J.W., Lee J.J., Lim J.S. Real-time NDE of steel cable using elasto-magnetic sensors installed in a cable climbing robot IAARC 2011. Proceedings of the 28th International Symposium on Automation and Robotics in Construction.

[14] Wang M.L. (2008). Long term health monitoring of post-tensioning box girder bridges. J. Smart Struct. Syst..

[15] Tang D., Huang S., Chen W., Jiang J. (2008). Study of a steel strand tension sensor with difference single bypass excitation structure based on the magneto-elastic effect. Smart Mater. Struct..

[16] Polar A., Indacochea J.E., Wang M.L. (2010). Sensing creep evolution in 410 stainless steel by magnetic measurements. J. Eng. Mater. Technol..

[17] Cao Y., Wang M.L. Cable stress monitoring for a cable stayed bridge. Proceedings of the 5th European Workshop on Structural Health Monitoring.

[18] Duan Y., Zhang R., Zhao Y., Or S., Fan K., Tang Z. (2011). Smart elasto-magneto-electric (EME) sensors for stress monitoring of steel structures in railway infrastructures. Appl. Phys. Eng..

[19] Duan Y., Zhang R., Zhao Y., Or S.W., Fan K., Tang Z. (2012). Steel stress monitoring sensor based on elasto-magnetic effect and using magneto-electric laminated composite. J. Appl. Phys..

[20] Wang M.L., Wang G., Zhao Y. (2005). Application of EM Stress Sensors in Large Steel Cables Sensing Issues in Civil Structural Health Monitoring.

[21] Esposito P. (2010). Structural Monitoring of the Cable Stayed Bridge on Adige River on North Trento—Rocchetta. Bachelor’s Thesis.

[22] Zhao Y., Wang M.L. Fast EM stress sensors for large steel cables. Proceedings of the SPIE 6934, Nondestructive Characterization for Composite Materials, Aerospace Engineering, Civil Infrastructure, and Homeland Security.

[23] Zui H. (1996). Practical formulas for estimation of cable tension by vibration method. J. Struct. Eng..

[24] Kim B.H., Park T., Shin H., Yoon T. (2007). A ccomparative study of the tension estimation methods for cable supported bridges. Steel Struct..

[25] Den Hartog J.P. (1985). Mechanical Vibrations.

[26] Kim B.H., Park T. (2007). Estimation of cable tension force using the frequency-based system identification method. J. Sound Vib..

[27] Robert C.P., Casella G. (2004). Monte Carlo Statistical Methods.

